# *Rickettsia parkeri* and *Candidatus* Midichloria sp. in *Amblyomma triste* ticks from protected areas of Buenos Aires Province (La Plata River Hydrographic Basin, Argentina)

**DOI:** 10.3389/fvets.2026.1841468

**Published:** 2026-06-23

**Authors:** Marina P. De Seta, Federico Krapp, Paula M. Díaz Pérez, Camila Giménez, Florencia Pastorino, Laura Alonso, Lorena Vico, Gustavo Martínez, María N. De Salvo, Gabriel L. Cicuttin

**Affiliations:** 1 Instituto de Zoonosis Luis Pasteur, Buenos Aires City, Argentina; 2 Departamento de Zoonosis Urbanas, Avellaneda, Buenos Aires, Argentina

**Keywords:** Midichloriaceae, one health, rickettsia, tick-borne diseases, Zoonosis

## Abstract

Ticks are important vectors of bacteria belonging to the order Rickettsiales, several of which are causes zoonotic diseases. *Amblyomma triste* is the main vector of *Rickettsia parkeri*, the etiological agent responsible for most cases of human spotted fever rickettsiosis in Argentina. However, information on the circulation of rickettsial agents in protected areas of the La Plata River Hydrographic Basin, a region that includes highly urbanized environments and frequent contact between wildlife, domestic animals and humans, remains limited. The aim of this study was to investigate the presence of rickettsial bacteria in *A. triste* collected in protected areas of Buenos Aires Province, (La Plata River Hydrographic Basin, Argentina). Between September and December 2021, ticks were collected by vegetation flagging in four protected areas and analyzed individually. Polymerase chain reactions (PCR) for detecting the genus *Rickettsia* was initially performed using a simple PCR to amplify a variablesized fragment of the 23S-5S *rRNA* intergenic spacer. An initial PCR was performed with primers for a 16S *rRNA* fragment for the Anaplasmataceae family. A total of 247 adult ticks were examined. Twelve specimens (4.9%) tested positive for *Rickettsia*, and sequence analysis confirmed their identity as *R. parkeri*. Positive ticks were detected in Ciervo de los Pantanos National Park and Campos del Tuyú National Park. In addition, two specimens yielded sequences corresponding to *Candidatus* Midichloria sp. These findings confirm the presence of *R. parkeri* in *A. triste* populations from protected areas of the La Plata River Hydrographic Basin and provide the first evidence of *Ca.* Midichloria sp. in this tick species in Argentina. The results highlight the need for continued surveillance of tick-borne microorganisms in protected areas located near densely populated regions, where human exposure to infected ticks may occur.

## 1 Introduction

Ticks (Acari: Ixodida) are hematophagous ectoparasites that are vectors of a wide diversity of important microorganisms to human and animal health, including bacteria belonging to the order Rickettsiales (1). The Rickettsiaceae family (order Rickettsiales, phylum Proteobacteria) include the genus *Rickettsia* composed of obligate intracellular Gram-negative bacterias, some of which are pathogenic to vertebrates and exhibit tropism for endothelial cells. The genus *Rickettsia* is divided into: the spotted fever group (SFG) transmitted by hard ticks (e.g., *Rickettsia rickettsii* and *Rickettsia. parkeri*); the typhus group transmitted by fleas and lice (*Rickettsia typhi* and *Rickettsia prowazekii*, respectively); the transitional group (TRG) (*Rickettsia felis* and *Rickettsia akari*), transmitted by fleas and mites; and the ancestral group (*Rickettsia bellii* and *Rickettsia canadensis)*, primarily transmitted by ticks (2).

The Anaplasmataceae family (order Rickettsiales, phylum Proteobacteria) includes the genera *Ehrlichia* and *Anaplasma*, among others (3). Obligate intracellular gram-negative bacteria of the genera *Ehrlichia* and *Anaplasma* reside in mature or immature hematopoietic cells, both in peripheral blood and in host tissues. They are transmitted by ticks, are etiological agents of diseases in dogs and other canids, humans, and ruminants (3). The Midichloriaceae family (Order Rickettsiales, Phylum Proteobacteria) is a newly emerging group of intracellular bacteria found in ticks, fleas, bed bugs, ciliates, amoebas, cnidarians, sponges, fish, and various vertebrates (4, 5). The endosymbiont *Candidatus* Midichloria mitochondrii, along with related species of the Midichloriaceae family, has been documented in ticks globally (4, 5).

*Amblyomma triste* (family Ixodidae, order Ixodida) belongs to the *Amblyomma maculatum* group, which also comprises *A. tigrinum* and *A. maculatum* (6), and is ecologically associated with wetlands and floodplains (1). The tick *Amblyomma triste* exhibits a broad host range that varies by life stage. Adults primarily parasitize large mammals, such as the marsh deer, capybaras, dogs and cattle, but are also known to bite frequently humans. In contrast, immature stages (larvae and nymphs) predominantly feed on small rodents. *Amblyomma triste* is one of the main vectors of *R. parkeri*, the agent of the highest incidence of human rickettsiosis in Argentina (7). Recently, an *Ehrlichia* sp. of unknown pathogenicity has been found in *A. triste* from Argentina (8).

The La Plata River Hydrographic Basin covers a large area of the coast of Buenos Aires Province.

(LRBP-BAP) which includes Argentina’s largest urban agglomeration, composed of the City of Buenos Aires and 40 municipalities in Buenos Aires Province (BAP). The LRBP-BAP contains numerous protected areas, many of which are in close proximity to urban or periurban zones and with frequent presence of domestic animals (9, 10). Few studies conducted more than a decade ago focused on *A. triste* and associated pathogens in protected areas of the LRBP-BAP (11–13), therefore, the aim of this research was to study the presence of rickettsial agents in adult specimens of *A. triste* collected in protected areas of the LRBP-BAP (Argentina).

## 2 Method

### 2.1 Study and sampling area

The LRBP-BAP is characterized by a sub-humid temperate climate with a pronounced estuarine influence that functions as a fundamental thermal moderator. The average annual temperature ranges between 16.7 °C and 18 °C, exhibiting a moderate thermal amplitude due to the vast body of water, with mean maximums of 22.2 °C and mean minimums of 10.7 °C. Precipitation is abundant, with annual records averaging between 1,000 mm and 1,200 mm distributed throughout the year, although occurring with greater intensity during the spring and summer months. Relative humidity remains high, with an annual average of approximately 79%.

Between September and December 2021, free-living ticks were collected using the flagging method in Ciervo de los Pantanos National Park (Campana; 34°13′37”S, 58°53′47”W), Avellaneda Municipal.

**Figure 1:**
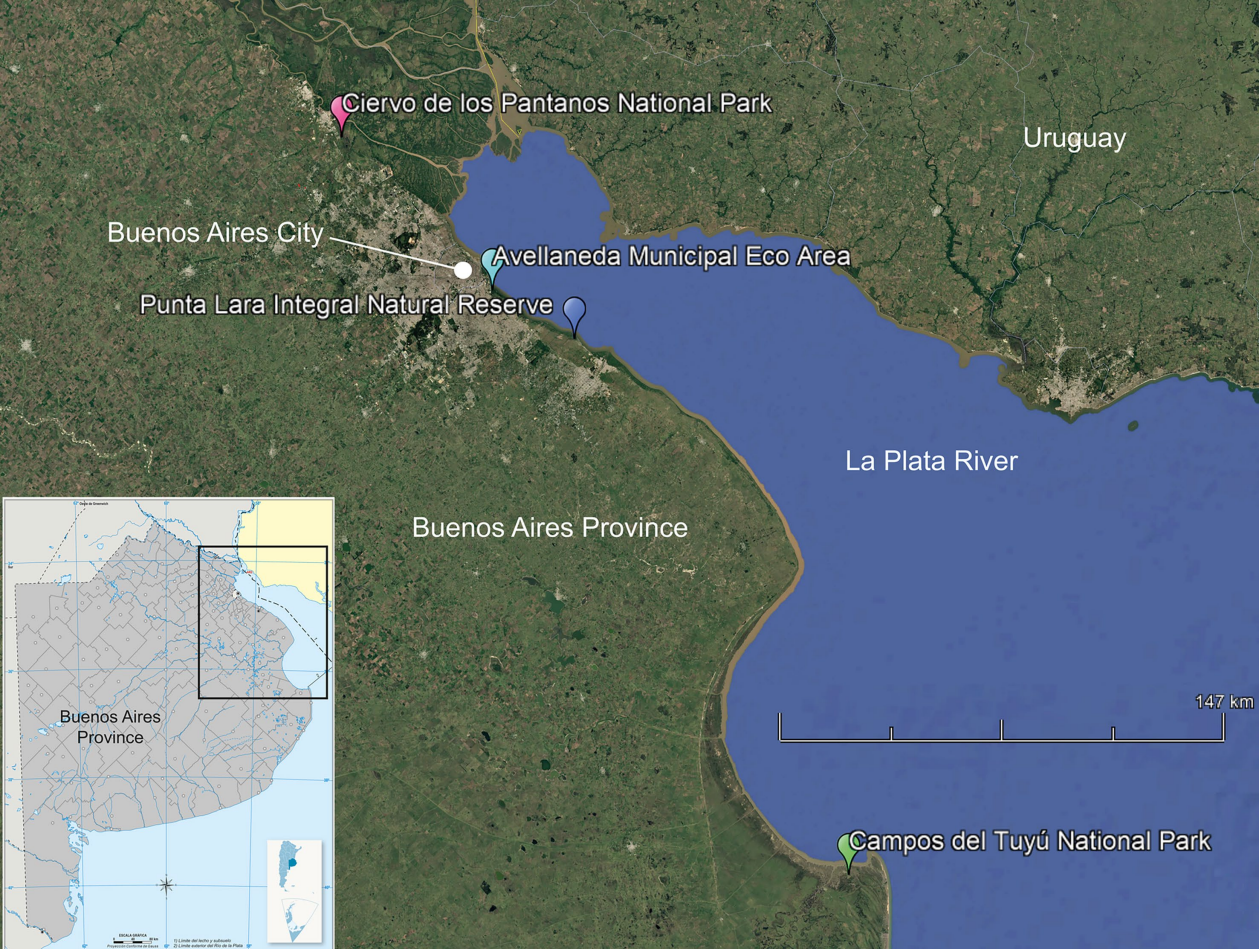
Sampling areas, Buenos Aires Provinces, La Plata River Hydrographic Basin (Argentina).

Eco Area (Avellaneda; 34°39′45”S, 58°19′4”W); Punta Lara Integral Natural Reserve (Ensenada;

34°47′38”S, 58°0′28”W) and Campos del Tuyú National Park (General Lavalle; 36°21′29”S, 56°51′27”W) (Figure 1). These reserves are characterized by diverse natural environments, including riparian forests, Pampas grasslands, and wetlands, and are located within or adjacent to urban and suburban areas (14). The ticks were preserved in 70% ethanol and subsequently identified following the taxonomic keys described by Nava et al. (1).

### 2.2 Molecular diagnosis

Tick specimens were processed individually, and DNA was extracted using the High Pure PCR Template Preparation Kit (Roche, Mannheim, Germany) according to the manufacturer’s instructions.

Detection of *Rickettsia* spp. was initially performed using a conventional PCR to amplify a variablesized fragment of the 23S-5S *rRNA* intergenic spacer (15). Molecular characterization was performed using PCR to amplify the *ompA* gene, which encodes the 190 kDa outer membrane protein of the genus Rickettsia (16). An initial PCR was performed with primers for a 16S *rRNA* fragment for the Anaplasmataceae family (17). This pair of primers has been routinely used to detect bacteria of this family; however, several studies have shown that they also detect a group of closely related Alphaproteobacteria within the order Rickettsiales, such as the Midichloriaceae family (18, 19). Nucleasefree water was used as a negative control for all PCR reactions. DNA from *Rickettsia massiliae* strain CABA and *Anaplasma centrale* served as a positive control for the PCRs for detection of *Rickettsia* and Anaplasmataceae, respectively.

### 2.3 Sequencing

Amplified PCR products were purified using the Wizard SV Gel and PCR Clean-Up System.

(Promega, Madison, WI, USA) and sequenced using a Genetic Analyzer 3,500 sequencer (Applied Biosystems, Foster City, CA, USA). The resulting sequences were edited using BioEdit Sequence Alignment Editor (20) with manual editing when necessary. The sequences were then compared with sequences deposited in GenBank using BLAST (https://www.ncbi.nlm.nih.gov/blast).

## 3 Results

Ticks were collected in the four protected areas and were identified as adults of the species *A. triste* (Table 1).

**Table 1:** Screening PCR results.

Study area *n*	PCR genus *Rickettsia* (%)	PCR Anaplasmataceae family (%)	
Ciervo de los Pantanos	97	7 (7.2%)	0 (0)
Avellaneda municipal eco area	9	0 (0)	0 (0)
Punta Lara Integral natural reserve	61	0 (0)	0 (0)
Campos del Tuyú National Park	80	5 (6.3)	2 (2.5)
Total	247	12 (4.9)	2 (0.8)

Twelve (12/247; 4.9%) specimens tested positive by PCR for a fragment of the 23S-5S intergenic *rRNA* spacer of the genus *Rickettsia*. All positive ticks were collected from Ciervo de los Pantanos National Park (7.2%) and Campos del Tuyú National Park (6.3%) (Table 1). Of the amplified products, 8/12 were sequenced (GenBank accession number PZ170526) resulting in 100% identity with each other and with previous findings of *R. parkeri*. A total of eight sequences were obtained; however, only four corresponded to the *ompA* gene, likely due to the higher sensitivity of the *23S rRNA* assay (15). The obtained sequences (GenBank accession number PZ150657) showed 100% identity with each other and with previous findings from *R. parkeri*.

By PCR for a fragment of the 16S *rRNA* for the Anaplasmataceae family, 0.8% (2/247) of the ticks tested positive (Table 1). The two amplified products were sequenced (GenBank accession number PZ148289), resulting in 100% identity with each other and with rickettsiae reported in *A. triste* from Uruguay, *Candidatus* Midichloria sp. in *Amblyomma tigrinum* from Brazil, and *C.* Midichloria sp. in *Amblyomma maculatum* from Colombia.

## 4 Discussion

This is the first report of *A. triste* in the Avellaneda Municipal Eco Area (Avellaneda) and in the Punta Lara Integral Natural Reserve (Ensenada), while there had been previous reports of this tick species in the Ciervo de los Pantanos National Park^1^ (13) and in the Campos del Tuyú National Park (11). *Amblyomma triste* is associated with wetlands and floodplains (1) and its distribution includes the LPRB (21, 22). Immature stages are found on rodents and, less frequently, on birds, while adults are mainly associated with larger wild and domestic mammals, such as marsh deer (*Blastocerus dichotomus*), capybaras (*Hydrochoerus hydrochaeris*), other deer species, wild carnivores, dogs, cats, cattle and horses, and frequently parasitizing humans (1).

Studies of pathogens associated with *A. triste* in the LPRB-BAP are scarce. Nava et al. (13) reported *R. parkeri* in 5.8% of *A. triste* collected from Ciervo de los Pantanos National Park, while Cicuttin and Nava (11) found *R. parkeri* in 7.0% of *A. triste* from Campos del Tuyú National Park, both infection levels similar to those found in the present study. *Rickettsia parkeri* was also reported in 5.0% of *A. triste* at Punta Indio (about 100 km from the Punta Lara Integral Natural Reserve) (12), while two studies have not found *Rickettsia* spp. in *A. triste* collected in the Costanera Sur Ecological Reserve of the Buenos Aires City, a protected area also located in the LPRB (23, 24). In another study, *Rickettsia* spp. were also not detected in *A. triste*, although the number of ticks studied was very low (25). It should be noted that numerous human cases of rickettsiosis caused by *R. parkeri* have been reported throughout the LPRB, including those associated with the Ciervo de los Pantanos National Park and the Campos del Tuyú National Park (12, 24–28).

Regarding the Anaplasmataceae family, this group of microorganisms was not detected in the present study. Previously, in areas of the Paraná Delta near Ciervo de los Pantanos National Park, *Ehrlichia* spp. (phylogenetically related to *Ehrlichia chaffeensis*) were reported in adult *A. triste*, both in questing ticks and from hosts (8, 29).

*Candidatus* Midichloria mitochondrii, as well as other related species of the Midichloriaceae family, have been reported in ticks worldwide (4, 5), but there are no previous reports of *Ca.* Midichloria sp. in *A. triste* from Argentina. Venzal et al. (19) detected this species (named “rickettsiales”) in 33% of *A. triste* ticks studied in Uruguay, a significantly higher infection rate than the one we found. The detection of *Candidatus Midichloria* sp. in ticks within urban areas, as reported by Cicuttin et al. (23), highlights the shifting ecological dynamics of the *Midichloriaceae* family and their role as potential biological markers for human-tick interactions. While primarily recognized as endosymbionts, their close phylogenetic relationship with known pathogens underscores the need for a One Health approach to monitor vector competence and zoonotic risk in urban environments.

In conclusion, *A. triste* is widely distributed throughout the LPRB, including areas where populations infected with *R. parkeri* have been identified. Given the public health importance of this pathogen, further studies are needed to improve our understanding of both the geographic distribution of this tick species and the microorganisms associated with it in Argentina.

## Data Availability

The raw data supporting the conclusions of this article will be made available by the authors, without undue reservation.

## Footnotes

1 Nava et al. (13) in their publication refer to the Otamendi Natural Reserve, which later became the Ciervo de los Pantanos National Park.
